# Targeted Protein-Specific Multi-Epitope-Based Vaccine Designing against Human Cytomegalovirus by Using Immunoinformatics Approaches

**DOI:** 10.3390/vaccines11020203

**Published:** 2023-01-17

**Authors:** Mohammed Ali Bakkari

**Affiliations:** Department of Pharmaceutics, College of Pharmacy, Jazan University, Jazan 45142, Saudi Arabia; mbakkari@jazanu.edu.sa

**Keywords:** epitope-based vaccine, human cytomegalovirus, immunoinformatics, MD simulations

## Abstract

Cytomegaloviruses are emerging pathogenic agents known to cause congenital disorders in humans. In this study, immune epitopes (CTL, B cell and HTL) were screened for highly antigenic target proteins of the Human Cytomegalovirus. These shortlisted epitopes were then joined together through suitable linkers to construct multi epitope-based vaccine constructs (MEVCs). The functionality of each vaccine construct was evaluated through tertiary vaccine structure modelling and validations. Furthermore, physio-chemical properties including allergenicity, antigenicity molecular weight and many others were also predicted. The vaccine designs were also docked with the human TLR-4 receptor to demonstrate the receptor specific affinity and formed interactions. The vaccine peptides sequences were also subjected to codon optimization to confirm the potential vaccines expression in *E. coli* hosts. Additionally, all the MEVCs were also evaluated for immune response (IgG and IgM) induction. However, further in vivo tests are needed to ensure the efficacy of these vaccine designs.

## 1. Introduction

Cytomegalovirus (also called CMV) is a well-known infectious agent in human history [1], which occurs in three forms including no infection, primary (seropositive) or latent infections with evolved strains [1]. All these types of infection are mostly under control with subclinical symptoms and viral replication is obstructed by the immune system. In contrast, CMV infections are more severe in immunocompromised patients with uncontrolled high viral loads observed in the urine. These symptoms may lead to viraemia and spreads to multiple organs causing several diseases including retinitis, pneumonitis, gastroenteritis or hepatitis [2]. CMV, as a common cause of intrauterine disease causing infectious agents are highly prioritized for vaccine development [3,4] in the world population. CMV has also a well-established link between lower socio-economic status and its higher prevalence [5]. This is reflected by higher prevalence of CMV in children born in developing countries as compared to developed countries [6]. Overall, there exists a strong correlation of cultural and socio-economic status with CMV prevalence.

According to the latest global estimates, CMV seroprevalance was reported the highest (about 90%) in the eastern Mediterranean region and the lowest (66%) in the European region [7]. Apart from this, women in the reproductive age have been also linked with increased seroprevalence of CMV [5,8]. In minority cases around the world, women during pregnancy with acquired primary level infections are at higher risk of CMV transmission and ultimate intrauterine infections in the new born [9,10]. Higher sero-prevalence of CMV is also linked with increasing age [11]. Similarly, reactivated CMV infections among the pregnant women belonging to countries with low sero-prevalence are also believed to result transmission into majority of new born babies [12]. Taken together, higher prevalence of CMV is aided by infections in pregnant women [13] or women with previous HIV infection [14]. These are the major factors contributing in global congenital or perinatal CMV burden. 

The genome of CMV comprises of UL (unique long) and US (unique short) regions. These UL and US regions are flanked by internal repeat and terminal sequences which regulates the viral replication by genome cleavage and packaging signals to facilitate the isomerization of the viral genome [15]. CMV virion diameter is about 230 nm [16] with a DNA core embedded inside the highly stable icosahedral capsid of 130 nm size. The larger size of CMV then other herpesviruses is a reflection of the larger genome. The envelope which surrounds the capsid comprises of viral glycoproteins which facilitates CMV attachment and cell entry [17]. Furthermore, the capsid composition includes four core proteins with involved role in CMV replication [18]. The nucleocapsid, enclosed by the tegument contains the DNA genome with two virion RNAs [17] In turn, the tegument is composed of 32 phosphorylated proteins surrounded in an envelope of lipid bilayer. The modified lipid bilayer envelope with insertions from virus-encoded glycoproteins, basically originates from the ER (endoplasmic reticulum) and Golgi Complex of the host cell [18]. The insertions of glycoproteins from the virus including gH, gB, gL, gM, and gN contained in its lipid bilayer are essential to viral DNA replication and are specific targets for neutralizing antibodies [18]. The integrated virion proteins also play vital roles in cell entry, egress and cell tropism during CMV infection.

Despite evaluation prospects of potent vaccines [19], further efforts are required in vaccine development as an effective and safe therapy for CMV infections. A developed vaccine will cop against cytomegalovirus infections and may prove as a cost effective therapy [20]. On the other hand, vaccine development is harbored by the CMV adapted diverse strategies in immune evasion and ability of causing reinfections. It also demonstrates CMV as a complex target in effective vaccine designing. This is reflected by termination of vaccine development process after a clinical candidate trial with potential protection against cytomegalovirus disease but not against CMV caused reinfections [21]. However, another candidate CMV gB based recombinant vaccine with adjuvant (MF59) only showed 50% protection at primary level and was considered to potentially decrease infections related to congenital and maternal CMV cases [22]. This vaccine, while administered during transplantation, also showed reduced risk of viraemia and enhanced protection through production of gB specific antibodies against the CMV virus [23]. Similarly, another clinical trial utilized two DNA plasmids in stem cell transplant patients and showed enhanced protection with increased detected number of cytokines and antibody secreting cells [24]. These findings also suggest the potential of CMV vaccines with the property of boosting immune response and thus protection against infections caused by CMV. Further efforts are needed in the design of improved vaccines with ability to enforce humoral immunity which may provide lifelong protection against CMV invasion. 

Reverse vaccinology and computational vaccine designing are time saving when compared to conventional approaches of vaccine development [25,26]. These approaches have been widely used to evaluate target-protein-specific and proteome-wide, effective, safe, and stable vaccine candidates [27,28,29]. In addition to therapeutic modalities and protection measures, it is critical to design effective vaccines against the congenital disorders in children. Herein, an immune-informatics approach was deployed against four highly antigenic target proteins. This was followed by immune epitopes screening to shortlist highly immunogenic peptides and design multi epitope-based vaccines against HCMV (Human Cytomegalovirus). These vaccine constructs were then modelled and evaluated for potential induction of immune response through molecular docking of the constructs with human TLR-4 receptor. Furthermore, codon optimization and immune simulations were also performed for confirmation of maximal expression in *E. coli* and potential immune response induction. Overall, the study offers four different target protein-specific vaccine designs as novel therapeutic candidates against Human Cytomegalovirus.

## 2. Methodology

### 2.1. Data Retrieval and Target Proteins Selection

**Figure 1:**
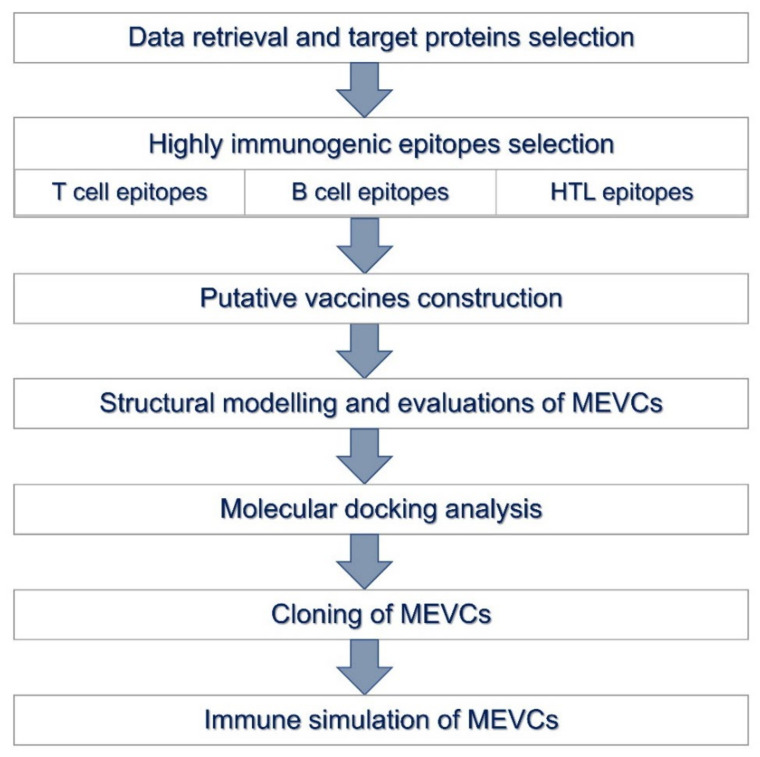
The overall workflow of the study design adapted in the construction of MEVCs.

The availability of novel biological resources [30,31] is helpful in the design of novel therapeutics against human pathogens [32,33]. The shortlisting of highly antigenic target proteins and epitope sequences predicted against each protein of the HCMV were retrieved from the previously developed online resource [31], whereas the antigenic and non-antigenic proteins for each specie were identified with a VaxiJen threshold scoring system [34]. The online available VaxiJen server (http://www.ddg-pharmfac.net/vaxijen/VaxiJen/VaxiJen.html; accessed on 11 September 2022) utilizes an alignment free, covariance-based approach with a focus on the amino acids properties [34]. Proteins were further subjected to allergenicity prediction analysis. The performed allergenicity check helps to ensure the prevention of possible allergic responses in the host [32] during the vaccine designing procedures. Algpred v. 2.0 (http://crdd.osdd.net/raghava/algpred/; accessed on 27 September 2022) server [33] was utilized to evaluate allergenicity status of the proteins. The input sequence was added as a single letter amino acid code while the selected prediction approach was an amino acid composition based SVM module [33]. The analyzed shortlisted epitopes for each target protein with potential efficacy are already available in the online resource for potential utility in vaccine construction. Further analysis was performed on the basis of shortlisted epitopes against each target protein from the HCMV proteome. All the retrieved information of the genomic data set (NCBI accession ID: NC_006273.2) and proteome (UniProt accession ID: UP000000938) of Human herpesvirus were collected and subjected to further analysis. The overall workflow of the performed study is shown in Figure 1.

### 2.2. Highly Immunogenic Epitopes Selection

The highly immunogenic epitopes selection was initiated by selection of protein specific CTL epitopes predicted with the utility of NetCTL-1.2 server (http://www.cbs.dtu.dk/services/NetCTL/; accessed on 1 October 2022) [35] and available online [36]. This was achieved through prediction of cytotoxic T lymphocyte (CTL) epitopes for each protein of all species by utilizing NetCTL 1.2 server (http://www.cbs.dtu.dk/services/NetCTL/; accessed on 1 October 2022) [37] and its further characterization on the basis of combined score. During analysis, the threshold to predict CTL epitopes was kept 0.75. The prediction of CD8^+^ or cytotoxic CTL epitopes was achieved using the NetCTLpan server considering 12 HLA supertypes (A1, A1, A3, A26, A24, B8, B7, B44, B39, B62, and B58) [35]. It involved various sequence processing steps, including MHC-I binding, TAP binding, and cleavage by proteasomes [35]. Immunogenic peptides restricted to MHCI were determined by “IEDB Class I Immunogenicity Tool” taking into account default parameters. Epitope ranking was done based on the binding score: where a higher binding score reflects higher probability of peptide to induce an immune response [38]. 

Similarly, B-cell epitope predictions was performed through ABCPred (http://crdd.osdd.net/raghava/ABCPred/; accessed on 6 October 2022) resource [39]. The predicted linear B cell epitopes were further filtered with a defined (0.5) threshold score. These scores were further used to rank epitopes; a higher score indicated that the peptide was more likely to elicit an immunological response. The B-cell epitopes prediction is essential to design efficient vaccines against pathogens. The online resource deployed in the analysis was used to map protein specific B-cell epitopes for each species. B-cell epitopes are mainly composed of dispersed amino acids that together interact with B-cell receptors. This combination of amino acids also helps the B Cell receptors in recognition of B Cell epitope as an antigen. [39]. The ABCPred server, after utilizing a partial recurrent neural network, results in an output with a single binary number as 1 or 0, which represents epitope and non-epitope, respectively. The well-trained recurrent neural network with a ranking system showed all the potential B Cell Epitopes generated based scores, where a higher score represents a higher probability of the input sequence as a potential epitope. 

### 2.3. Putative Vaccines Construction

The shortlisted epitopes for each target protein from the HCMV proteome were further characterized on the basis of suggested higher binding affinity and grouped as suitable candidates for vaccine designing. The highly immunogenic epitopes were combined in a sequential way with the addition of suitable linkers. The final vaccine construction procedure involved addition of adjuvant, followed by T cell and B cell epitopes joined through different linkers including AAY and KK, respectively [40,41]. Furthermore, another EAAK linker was added to the N terminal of vaccine construct to attach the adjuvant (Human Beta Defensin-2) to the N terminal of the vaccine construct to enhance immunogenicity [42]. Next, the antigenicity of vaccine constructs vital to provoke robust immune response was predicted. Herein, the VaxiJen server [34] was utilized to investigate the antigenic potential of vaccine constructs with a default (0.4) threshold value. This was followed by allergenicity status evaluation by utilizing AlgPred server with a high (85%) estimated accuracy rate [43]. 

### 2.4. Structural Modelling and Evaluations of MEVCs

Furthermore, the 3D structures for all the vaccine constructs were projected by utilizing the Robetta web server (https://robetta.bakerlab.org/; accessed on 15 October 2022) [44] by choosing the de novo structure prediction method. In this procedure, the submitted sequences are scanned through domain-based initial recognition to forecast structure. This is followed by 3D modelling of submitted sequences depending on the availability template structures in the database. Upon availability of matching templates, comparative modelling is performed. In contrast, if no template is available then the 3D structures are modelled through de novo approach. Furthermore, several physio-chemical properties, including vital parameters to verify the feasibility of experimental processing, were explored. This analysis was performed by utilizing the web server called ProtParam (https://web.expasy.org/protparam/; accessed on 20 October 2022) [45] for all of the individual vaccine constructs. Moreover, different physicochemical properties of the designed vaccine candidates were also evaluated by utilizing an online server ProtParam (http://web.expasy.org/protparam/; accessed on 20 October 2022) [46]. These predictions help in determination of molecular weight, amino acid composition, in vitro half-life, aliphatic index, in vivo half-life, GRAVY and theoretical PI of the vaccine constructs. Moreover, for evaluations of transmembrane regions in the primary sequences of designed each MEVC, an online server “DAS”-Transmembrane Prediction server (https://tmdas.bioinfo.se/; accessed on 23 October 2022) was utilized [47]. 

### 2.5. Molecular Docking and Interaction Analysis

Using the ab initio free docking approach from HDOCK server [48], the designed vaccines were docked with the human TLR4 (Human Toll-like receptor-4). This server utilizes both template- and docking-based binding models of two molecules and allows its interactive visualization. Moreover, mmGBSA analysis were also performed for each of the docking complexes using HawkDock server [49]. The server employs a hybrid docking method setting it apart from other similar servers. The HawkDock server provides experimental information on the protein–protein binding site and small-angle x-ray drip in a quick and authentic manner [49]. We docked the MD2 protein as a native ligand with TLR4. Furthermore, PDBsum server [50] was utilized to analyze the interaction patterns between the docked molecules. The vaccine design was modeled using Robetta before protein–protein docking, and the TLR4 structure was acquired from RCSB [51]. The structures were prepared before the docking by removing water molecules, heteroatom and other atoms. 

### 2.6. Cloning of MEVCs

We used the Java codon adaptation tool (JCat tool) for codon optimization after reverse translation of the MEVC constructs [52]. The JCat program is also used to ensure that the vaccination sequence is expressed at a high level in an appropriate vector. Three choices, such as bacterium ribosomal binding sites, Rho-independent transcription termination and restriction enzyme cleavage sites, were selected in this tool. JCat determines the GC content and CAI score of the constructed vaccine to optimize the reversely transcribed vaccine construct in a bacterial expression system [53]. The EcoRI and XhoI, restriction enzymes were manually inserted into the vaccine sequence, and the sequence was subsequently cloned onto the pET-28a (+) plasmid using Snapgene software [54]. 

### 2.7. Immune Simulation of MEVCs

To create an immunogenic profile of the designed vaccines, we performed the immune simulation by using the online server called C-ImmSim (http://150.146.2.1/C-IMMSIM/index.php; accessed on 1 November 2022) [55]. C-ImmSim is a dynamic agent-based simulator for assessing the immunological responses of the body to antigen. C-ImmSim uses machine learning techniques and the specific scoring matrix PSSM to predict immune interactions and epitopes. Following the submission of the designed vaccine to the online server with the default simulation settings, the production of antibodies, interferon, and cytokines were measured; however, the same server was also used to check the reactions of both Th1 and Th2 [56].

## 3. Results

### 3.1. Target proteins Selection for Potential Epitopes Prioritization in Vaccine Construction

The characterization of immunogenic protein peptides is a prerequisite to design therapeutic strategies against human infecting viruses [57,58]. Firstly, target proteins were prioritized from the whole proteome HCMV based on highly antigenic scores. This included one of the five HCMV-encoded glycoprotein called US3 which interferes with MHC class I antigen presentation, thus hindering viral clearance by cytotoxic T lymphocytes (CTLs) [59]. Moreover, the uncharacterized UL15A and UL41A were also shortlisted based on higher antigenicity. Similarly, UL40 which promotes efficient cell surface expression of the non-classical MHC-I molecule was also studied [60]. Here, we utilized the T cell and B cell epitopes retrieved against each highly antigenic target proteins from the previously developed online resource for CMV [31]. This helped in the characterization and selection of suitable epitopes for the protein-specific vaccine design against CMV. The details of shortlisted proteins and selected epitopes included in MEVC designing procedure are given in Table 1. 

### 3.2. Multi-Epitope-Based Vaccine Constructs 

**Figure 2:**
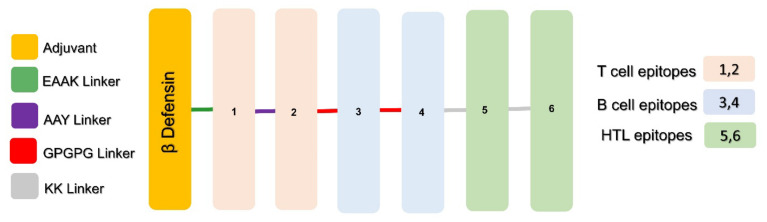
Showing the topographical organization of adjuvant, epitopes, and linkers in each of the designed MEVC.

The different shortlisted epitopes based on immunogenic properties were then subjected to MEVC designing procedures. This was performed with the addition of EAAK linker to combine the non-toxic adjuvant called human beta defensin-2 (hBD-2) at the N terminal domain of each protein-specific vaccine design. The hBD-2 possesses the property of self-production with expression levels which enable inducing a robust immune response against the attached antigen. Using this approach, MEVCs were designed for the highly immunogenic target proteins of HCMV. The shortlisted epitopes were then used in the full-length MEVC designs and joined together using different linkers (Figure 2). The details of full length (165 amino acids) MEVC designed against each target protein with antigenic properties are given in Table 2. 

These adjuvants linked immune epitope-based vaccine constructs are capable of expressing the sequence of the most important pathogen protein antigens in DNA plasmids. Advanced strategies involving virus-like particles (VLPs) [61,62] and nanoparticles [63,64] have been used as vehicles for delivering multi-epitope vaccines. Upon host-delivery of the subunit peptide vaccines, a conformation-dependent way is followed by antibodies in targeting these antigens [65]. This is facilitated by interactions of the antigen residues contained in the immune epitopes with side chains involved in establishing direct contact with the antibody combining site. This antibody recognition depends on the globular fold and incorporate residues from many secondary structural components [66]. In the case of T-cell responses, specific regions of an antigen also result in the expansion of T-cells. Importantly, immunodominance related issues are important factors considered for a peptide-based vaccine which focuses on a limited number of critical epitopes [67]. This implies the importance of selecting highly antigenic epitopes for induction of better immune responses while designing peptide-based vaccine constructs.

### 3.3. Physiochemical Properties Evaluations

The vaccine constructs were also subjected to analysis through ProtParam server to verify several physiochemical parameters vital to confirm the stability of the vaccine designs. These parameters included the calculation of molecular weight, theoretical pI and other features suggesting feasibility and stable structure of the vaccine for further experimental designs. Moreover, the demonstrated aliphatic index (thermo-stability) and GRAVY (Grand average of hydropathicity) were also explored, respectively. The different physiochemical properties explored for proteome-wide multi-epitope vaccines were predicted for MMVs and presented in Table 3. Furthermore, the potential soluble expression of each vaccine construct in *E. coli* was also validated through screening of transmembrane regions (Figure S1 and Table S1) based on the analysis of primary sequences. 

The ProtParam server estimated the molecular weight of all the MEVCs was between 17–18 KDa, showing the optimal range for proteins expression. Similarly, the theoretical protrusion index (PI) was about 9 to 9.82. Moreover, the predicted in vivo half-life for all the MEVCs in *E. coli* was >10 h. The vaccine’s thermostable nature was also confirmed with aliphatic index ranging from 70 to 74 for the different MEVC’s. Furthermore, the protein’s lower GRAVY values implied that it is hydrophilic and may have improved interactions with adjacent water molecules.

### 3.4. Structural Modelling and Validations of the Designed MEVCs

**Figure 3:**
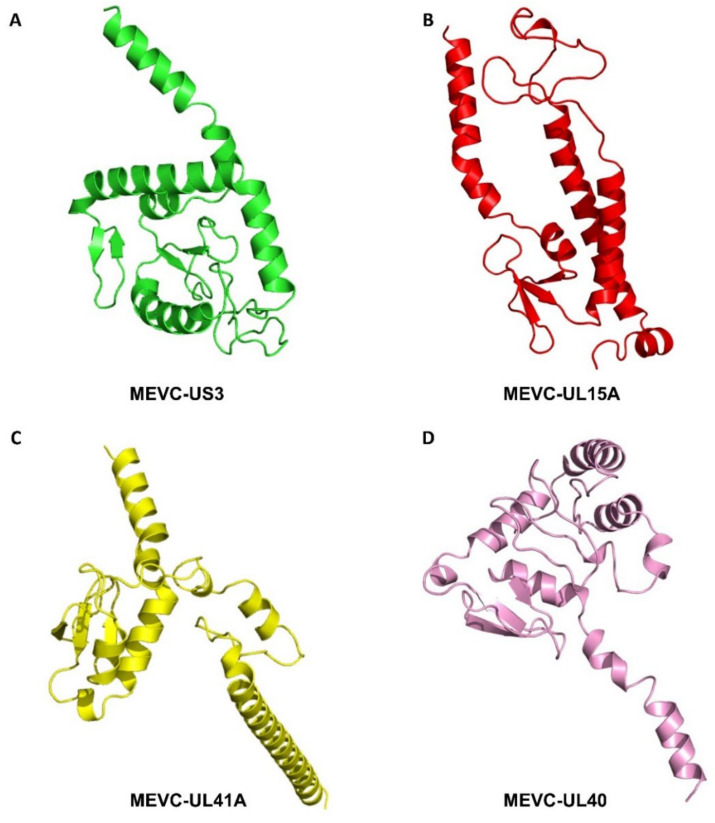
(**A**–**D**) Represents the predicted 3D structures of final multi-epitope-based vaccine designs for HCMV proteins.

All the MEVC structures were modelled using the Robetta server which utilizes comparative modeling or de novo sequence based structural prediction methods. This server is capable of generating efficient structural models by analyzing potent domains in the amino acid sequences. This follows a template-based matching protein structural models to generate a comparative model. However, it also utilizes the ab initio method for novel proteins due to non-availability of matching structures. By utilizing the Robetta-Fold module, we generated several 3D structural models for each of the constructed MEVCs against HCMV. The several generated models were then visualized and best models predicted for each MEVC was refined in the PyMOL software. Moreover, all the modelled structures for each MEVC were also validated through ProSA-Web analysis and Ramachandran Plots (Figure S2). The evaluated and shortlisted 3D MEVC model structures for each protein target specific vaccine design against HCMV are shown in Figure 3.

### 3.5. Molecular Docking and Interactions of MEVCs with Human TLR4

**Figure 4:**
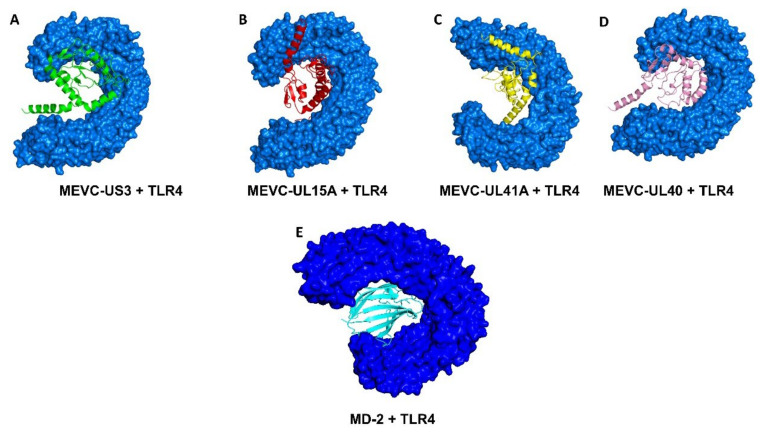
(**A**–**E**) Represents the docking complexes of final multi-epitope-based vaccine designs with human TLR-4 for each of the target HCMV protein.

Similarly, docking and interaction analysis was performed for the four target proteins of HCMV and native ligand (MD-2) with TLR-4. Among the HDOCK, server generated top ten docking models, and only the best of MEVC-TLR4 complexes was shortlisted on the basis of docking score obtained for each target protein of HCMV as shown in Figure 4.

The HDOCK and PDBsum-generated interaction analysis also revealed that for MEVC-US3, the docking score was −302.71 kcal/mol while five hydrogen bonds and no salt bridges were reported. Similarly, the MEVC-UL15A also formed six hydrogen bonds and one salt bridge with the docking score −346.35 kcal/mol. Moreover, in MEVC-UL41A complex with a docking score of −336.44; the total number of hydrogen bonds were seven and number of salt bridges were two. Finally, with the formation of 8 hydrogen bonds and 1 salt bridge, the docking score for MEVC-UL40 was −340.42 kcal/mol. Moreover, the free energy calculations were also performed for all the docking complexes by using the MM/GBSA approach (Table S2). The results decisively suggest that the designed vaccine candidates robustly interact strongly with the human immune receptor TLR4 (Figure S3). The MD2-TLR4 docking results are presented in Figure 4E. The docking score was obtained to be −247.50 which is lower than the designed vaccines, thus showing the potential of our vaccine candidates. The docking scores acquired for each of the docking complex of HCMV target proteins with human TLR4 are given in Table 4. 

### 3.6. Codon Optimization and In silico Cloning 

**Figure 5:**
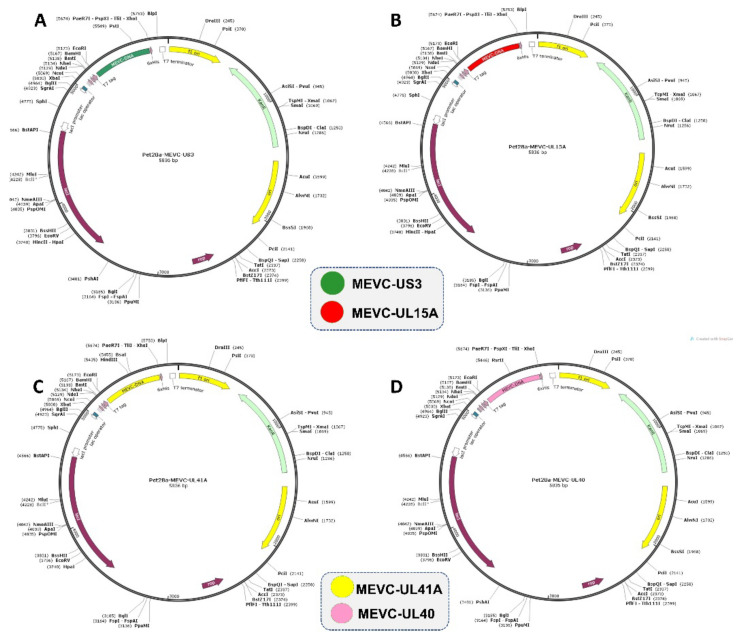
Represents the in silico cloning designs of the final multi-epitope-based vaccine against each specific target protein of HCMV. The different inserts in the same vector (Pet28a+) are represented with specific colors (Green, Red, Yellow and Pink).

Similarly, all the improved DNA sequences for each vaccine construct against HCMV target proteins were obtained. The set of restriction enzymes XhoI and ECORI were selected for cloning of the gene sequence into pET28a (+) vector. The software package Snapgene was utilized for the insertion of MEVC-DNA sequences into the plasmids and cloning designs were obtained. Finally, in silico clones were obtained for each target protein of HCMV as shown in Figure 5. The codon adaptation index (CAI) values were also calculated through the JCat server for each of the vaccine constructs. The higher calculated CAI scores in the range (0.98–1) and optimal percentage of GC contents (51 to 53%) was indicative that the vaccine protein is expressed at a high level in *E. coli*. 

### 3.7. Immune Simulation of the Proposed MEVCs 

**Figure 6:**
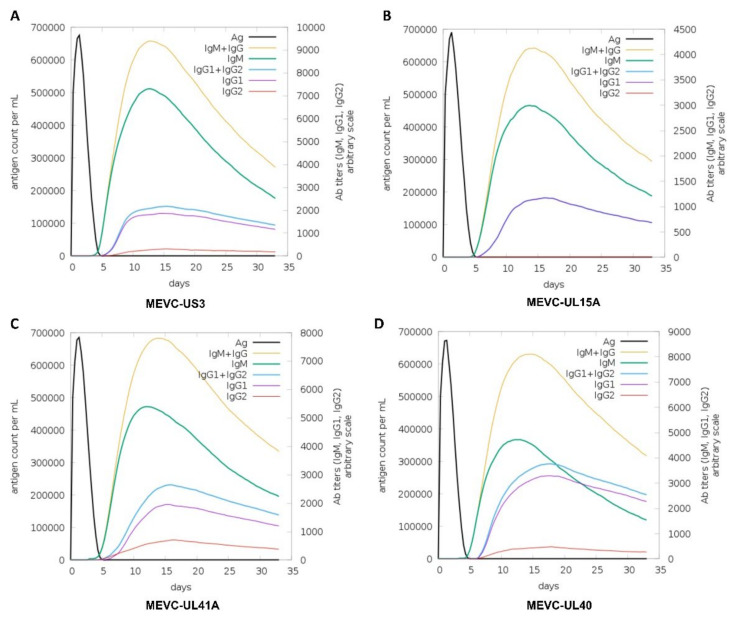
Represents the immune simulation graphs showing plotted antigen count/mL/day against Ab titers for each of the antigenic vaccine (MEVC) designs. (**A**–**D**) Represents the immune simulation graph for vaccines designed against each target protein of HCMV.

Similarly, immune-simulation analysis was performed for each MEVC designed and graphs were obtained for each target protein of HCMV (Figure 6). It can be observed that the injected antigen after achieving the highest antigen counts at day 5 were slowly neutralized till day 15 for all the vaccine constructs. For “MEVC-US3”, a higher ratio (9000 au/mL of antibody (IgM + IgG) titers) was observed (Figure 6A). Similarly, higher antibody titers were observed for “MEVC-UL15A”, “MEVC-UL41A” and “MEVC-UL40” ranging from 4000–8000 au/mL (Figure 6B–D). This trend was followed by IgM specific antibody titers of about ≥2500 to 7000 au/mL for the different MEVCs. Upon injection, these results reflected the immunogenic potential of each designed vaccine which triggers a robust immune response. Consequently, the designed vaccine candidates may trigger the production of protective immunity against the human pathogenic CMV after potential evaluation in further in vivo and in vitro models.

## 4. Conclusions

In conclusion, the reverse vaccinology approach has been utilized to shortlist putative vaccine epitopes including CTL and B cell epitopes for each of the target protein from HCMV proteome. Firstly, highly immunogenic epitopes were screened for each target protein of HCMV and then joined together through suitable linkers to construct a target-specific vaccine. It was followed by multi-epitope-based vaccine designing against four highly antigenic proteins of HCMV. The constructed MEVCs have been modelled and evaluated for potential immunization against HCMV. The vaccine designs were also docked with the human TLR-4 receptor to demonstrate the receptor specific affinity and formed interactions. Additionally, by subjecting the peptides sequences to codon optimization, the potential vaccine expression in the *E. coli* host were also evaluated. The current work identifies new and valuable epitope candidates with potential utility in future vaccine development which may provide protection against congenital infections caused by human cytomegalovirus. The results of our investigations suggest a powerful immune response induction on the administration (injection of purified protein or immunogen in viral vector) of the proposed vaccine constructs. The performed research also assists the path for processing of epitope-based vaccines against HCMV experimentally. The main limitation of this study is experimental validation required for the final vaccine candidates to confirm its immune reinforcement potential and clinical use. 

## Figures and Tables

**Table 1:** Shows the details of target proteins and the amino-acids sequences of different epitopes (CTL, B cell and HTL) used in the design of MEVCs against the four highly antigenic proteins of HCMV.

**Index**	Target Protein	Description/ Function	Antigenicity Score	CTL Epitopes	B Cell Epitopes	HTL Epitopes
1	membrane glycoprotein US3	glycoprotein/ hinders viral clearance by cytotoxic T lymphocytes (CTLs)	0.83	YSSQTINWY, SSQTINWYL	SGEQYHHDERGAYFEW, SQTINWYLQRSMRDDNST	FRTLLVYLFSLVVLV, GEQYHHDERGAYFEW
2	protein UL15A	uncharacterized	0.71	LCSWLAMRY, RTDHQKADI	GALICGSGTRRGSGAN, ACTRTDHQKADIGLWF	MFLVFGLCSWLAMRY, TRTDHQKADIGLWFM
3	protein UL41A	uncharacterized	0.71	RTANSTAGY, FCSTLKAFY	ETTVWEKRRMESDTDF, LFCRTANSTAGYVDMN,	ITKIMLARRKARAMV, WLVGVGIFMAGGFIA
4	membrane glycoprotein UL40	glycoprotein/ promotes efficient cell surface expression of the non-classical MHC-I molecule	0.7	ALGSFSSFY, TSSNTVVAF	HQDCPAQTVHVRGVNE, VDGISCQDHFRAQHQD,	TVGILALGSFSSFYS, CMRIRSLLSSPVETT

**Table 2:** Shows the full-length designed MEVCs against the four highly antigenic proteins from HCMV.

**Index**	**Vaccine-Target Protein**	Protein-Specific MEVC Constructs	Antigenicity Scores
1	MEVC-US3	MRVLYLLFSFLFIFLMPLPGVFG GIGDPVTCLKSGAICHPVFCPRRYKQIGTCGLPGTKCCK KPEAAKYSSQTINWYAAYSSQTINWYLGPGPGSGEQYHHDE RGAYFEWGPGPGSQTIN WYLQRSMRDDNKKFRTLLVYLFSLVVLVKKGEQYHHDERG AYFEW	0.7
2	MEVC-UL15A	MRVLYLLFSFLFIFLMPLPGVFG GIGDPVTCLKSGAICHPVFCPRRYKQIGTCGLPGTKCCK KPEAAK LCSWLAMRYAAYRTDHQKADIGPGPGGA LICGSGTRRGSGANGPGPGACTRTDHQKADIGLWFKK MFLVFGLCSWLAMRYKKTRTDHQKADIGLWFM	0.59
3	MEVC-UL41A	MRVLYLLFSFLFIFLMPLPGVFG GIGDPVTCLKSGAICHPVFCPRRYKQIGTCGLPGTKCCK KPEAAK RTANSTAGYAAYFCSTLKAFYGPGPGETTVWE KRRMESDTDFGPGPGLFCRTANSTAGYVDMNKK ITKIM LARRKARAMVKK WLVGVGIFMAGGFIA	0.52
4	MEVC-UL40	MRVLYLLFSFLFIFLMPLPGVFG GIGDPVTCLKSGAICHPVFCPRRYKQIGTCGLPGTKCCK KPEAAK ALGSFSSFYAAYTSSNTVVAF GPGPGHQDCPAQTVHVRGVNEGPGPGVDGISCQDHFRAQ HQDKK TVGILALGSFSSFYSKKCMRIRSLLSSPVETT	0.58

**Table 3:** Represents the physiochemical properties of the designed MEVCs against four target proteins of HCMV.

Index	Vaccine Name	Molecular Weight (Kilo Daltons)	Theoretical pI	Negatively Charged Residues (Asp + Glu):	Positively Charged Residues (Arg + Lys)	Total Atoms	Aliphatic Index	Hydropathicity (GRAVY)
1	MEVC-US3	18 kds	9	12	18	2627	70.30	−0.278
2	MEVC-UL15A	18 kds	9.67	8	23	2542	73.39	0.024
3	MEVC-UL41A	17.9 kds	9.82	8	24	2542	72.18	0.122
4	MEVC-UL40	17.6 kds	9.25	8	17	2475	74.42	0.093

**Table 4:** Represents docking scores and number of formed interactions between the designed MEVCs and human TLR4.

Index	Vaccine Name	TLR Name	Salt Bridges	Hydrogen Bonds	Non Bonded Contacts	Docking Score
1	MEVC-US3	TLR4	0	5	151	−302.71
2	MEVC-UL15A	TLR4	1	6	200	−346.35
3	MEVC-UL41A	TLR4	2	7	225	−336.44
4	MEVC-UL40	TLR4	1	8	234	−340.42
5	MD-2	TLR4	1	5	158	−247.50

## Data Availability

The data presented in this study are available within the article.

## Supplementary Materials

The following supporting information can be downloaded at: https://www.mdpi.com/article/10.3390/vaccines11020203/s1, Figure S1: Showing the predicted trans-membrane segments predicted for each MEVC designed against HCMV. (A–D) represents the residues above the threshold (dotted line) as potential transmembrane regions characterized for each of the target vaccine i.e., MEVC-US3, MEVC-UL15A, MEVC-UL41A and MEVC-UL40, respectively. Figure S2: Showing structural validation of each MEVC designed against HCMV through Prosa-web analysis and Ramachandran plot. (A–D) represents the Prosa-Web analysis and Ramachandran plot of MEVCs designed against each target protein i.e., US3, UL15A, UL41A and UL40, respectively. Figure S3: Showing the interaction patterns of each MEVC designed against HCMV with human TLR4. (A–D) represents the formation of different interactions formed between each MEVC with human TLR4 characterized for each of the target protein i.e., US3, UL15A, UL41A and UL40, respectively. Table S1: Represents the identified transmembrane segments in the designed MEVCs against each target protein. Table S2: Represents the docking free energy calculations of the designed MEVCs with human TLR4.
