# Supplementary material for: Targeted Protein-Specific Multi-Epitope-Based Vaccine Designing against Human Cytomegalovirus by Using Immunoinformatics Approaches
Source: Vaccines (Basel). 2023 Jan 17;11(2):203. doi: 10.3390/vaccines11020203 (PMC9959080; doi:10.3390/vaccines11020203)
Supplement: Supplementary file 1 [file vaccines-11-00203-s001.zip › vaccines-2127917-supplementary.pdf]

Table S1: Represents the identified transmembrane segments in the designed MEVCs against each target protein.

Docking complex	VDW	ELE	GB	SA	TOTAL
**MEVC-US3 + TLR4**	-157.58	-1050.5	1175.52	-18.94	-51.51
**MEVC-UL15A + TLR4**	-192.78	-2577.86	2724.59	-24.07	-70.13
**MEVC-UL41A + TLR4**	-154.1	-3210.29	3295.35	-18.75	-87.79
**MEVC-UL40 + TLR4**	-151.84	-1619.83	1740.97	-19.02	-49.72
**MD-2-TLR4**	-95.74	-1230.13	1299.62	-14.74	-40.99

Table S2: Represents the docking free energy calculations of the designed MEVCs with human TLR4.

Vaccine name	Start Residue	Stop Residue	Length	Transmembrane segments
**MEVC-US3**	4<br>136	25<br>148	22<br>13	2
**MEVC-UL15A**	4<br>133	22<br>144	18<br>12	2
**MEVC-UL41A**	5<br>152	23<br>160	19<br>9	2
**MEVC-UL40**	5<br>136	24<br>148	20<br>13	2

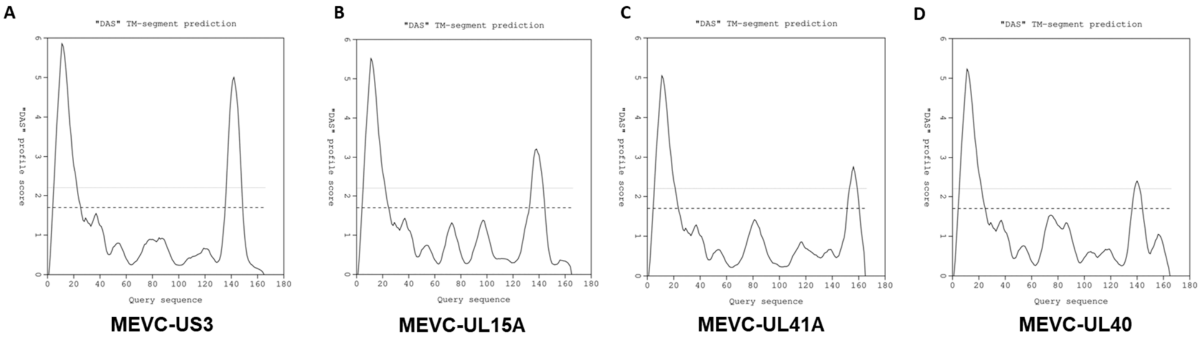

Figure S1: Showing the predicted trans-membrane segments predicted for each MEVC designed against HCMV. **(A-D)** represents the residues above the threshold (dotted line) as potential transmembrane regions characterized for each of the target vaccine i.e., MEVC-US3, MEVC-UL15A, MEVC-UL41A and MEVC-UL40, respectively.

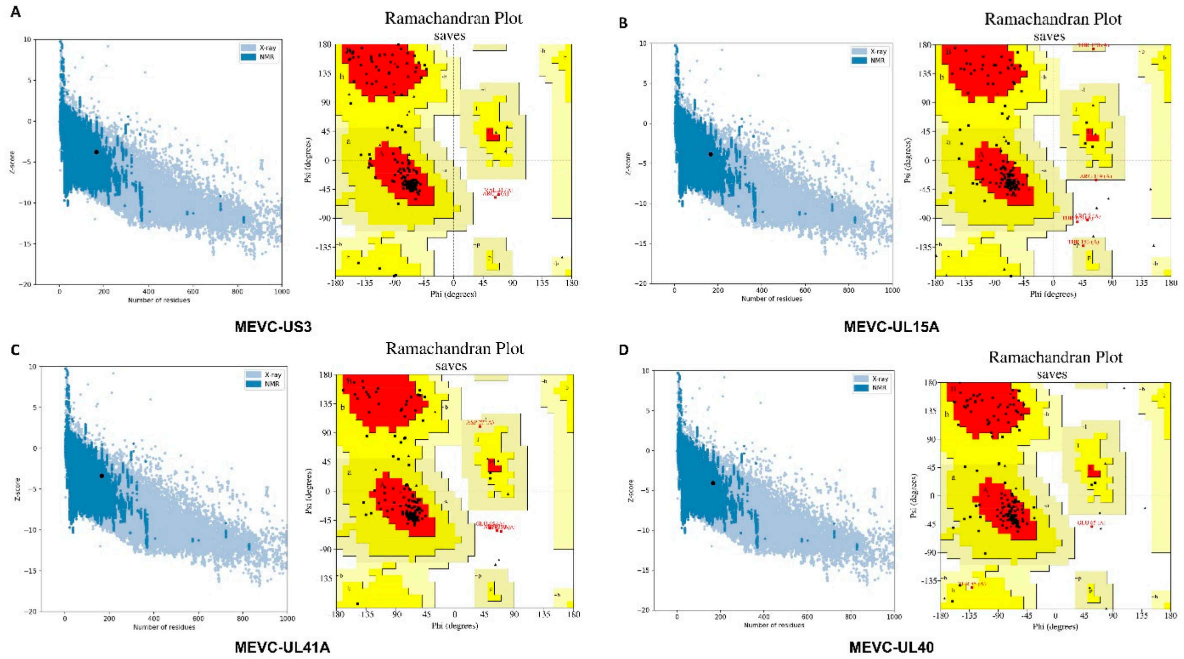

Figure S2: Showing structural validation of each MEVC designed against HCMV through Prosa-web analysis and Ramachandran plot. **(A-D)** represents the Prosa-Web analysis and Ramachandran plot of MEVCs designed against each target protein i.e., US3, UL15A, UL41A and UL40, respectively.

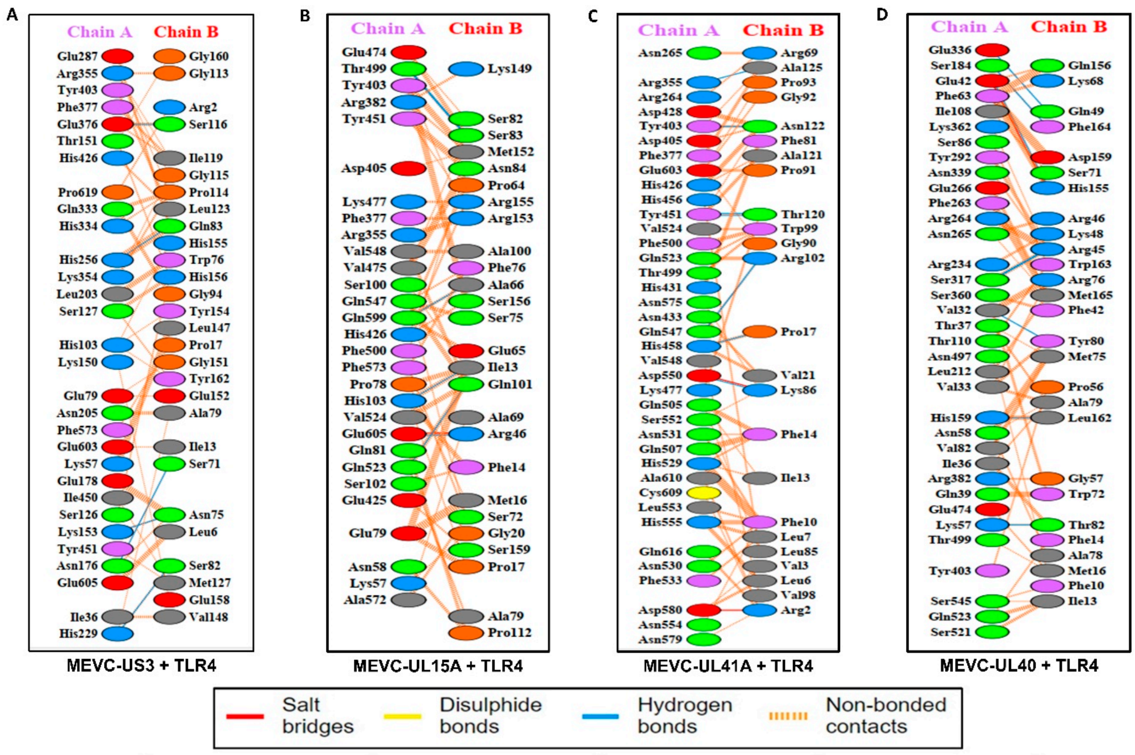

Figure S3: Showing the interaction patterns of each MEVC designed against HCMV with human TLR4. **(A-D)** represents the formation of different interactions formed between each MEVC with human TLR4 characterized for each of the target protein i.e., US3, UL15A, UL41A and UL40, respectively.
